# How to get the best deal

**DOI:** 10.7554/eLife.09919

**Published:** 2015-08-11

**Authors:** Kelsey JRP Byers, Florian P Schiestl

**Affiliations:** Institute of Systematic Botany, University of Zurich, Zurich, Switzerland; Institute of Systematic Botany, University of Zurich, Zurich, Switzerlandflorian.schiestl@systbot.uzh.ch

**Keywords:** *Nicotiana attenuata*, *Manduca sexta*, *Hyles lineata*, *Archilochus alexandri*, *Manduca quinquemaculata*, pollination, other

## Abstract

Floral scents and nectar attract both pollinators and other animals that may reduce the plant's fitness, and therefore put flowering plants in a challenging situation.

**Related research article** Kessler D, Kallenbach M, Diezel C, Rothe E, Murdock M, Baldwin IT. 2015. How scent and nectar influence floral antagonists and mutualists. *eLife* **4**:e07641. doi: 10.7554/eLife.07641

**Image** Coyote tobacco flowers being visited by a hawkmoth

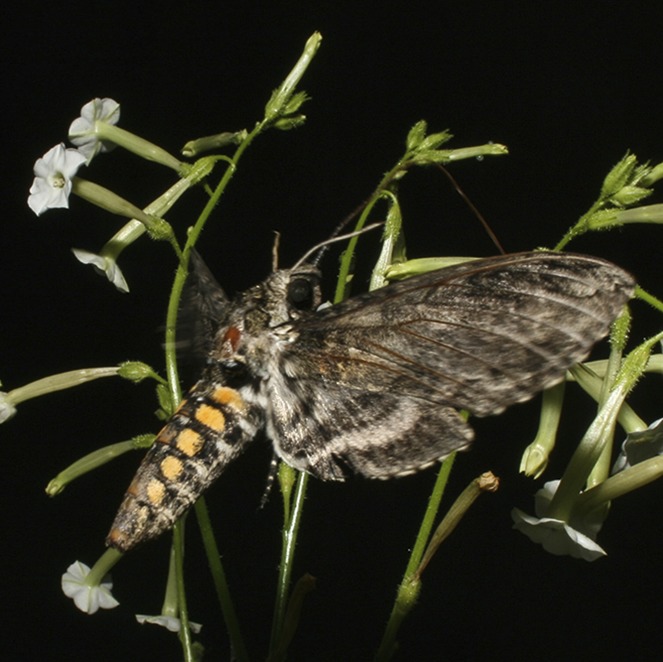

Most flowering plants rely on animals to spread their pollen. However, plants that rely on easily perceived signals, such as brightly coloured petals and floral scents, to attract pollinators are also advertising themselves to other animals that cause damage. These so-called ‘floral antagonists’ include animals that eat plant tissues (herbivores and florivores) and animals that steal nectar and pollen without helping with pollination.

These different interactions mean that flowering plants are subjected to a range of selection pressures. However, while most published research has focused on seemingly mutually beneficial relationships, little is known about how a plant can attract beneficial visitors and at the same time hide from floral antagonists that might cause harm. Plants attempt to address these challenges in multiple ways to maximize their fitness (Galen and Cuba, 2001; Chen et al., 2009; Kessler et al., 2008, 2013; Schiestl et al., 2014). The picture is complicated further when a single animal can act as both a pollinator and a floral antagonist (e.g., by wasting pollen, robbing nectar, or switching roles at different life stages; Adler and Bronstein, 2004). This puts the plant in a difficult situation, since the animal is responding to the same signals despite playing different roles. Any attempt by the plant to change its strategy to avoid the antagonist will also reduce pollination.

Now, in eLife, Danny Kessler, Ian Baldwin and colleagues at the Max Planck Institute for Chemical Ecology have assessed the roles played by a range of pollinator and antagonist species to develop a more complete picture of plant-pollinator interactions (Kessler et al., 2015). The MPI team used coyote tobacco, *Nicotiana attenuata*, to investigate how floral scent and nectar affect this plant’s interactions with three of its pollinators: a hummingbird (*Archilochus alexandri*) and two hawkmoths (*Hyles lineata* and *Manduca sexta*). The first two species appear to act as mutualists, trading pollination for a nectar reward. However, *M. sexta* plays contrasting roles; the adult moths pollinate the flowers, but the females also lay eggs on plants and the caterpillars eat the leaves (Figure 1).

**Figure 1.**
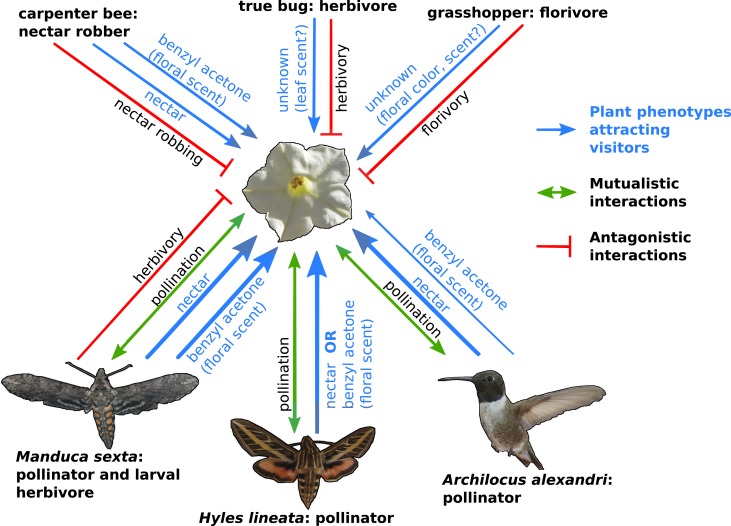
The complexity of plant-pollinator interactions. Coyote tobacco (*centre)* interacts with pollinators (the three studied by Kessler et al. are shown) and with floral antagonists (three examples are shown at the top of the figure) in a variety of ways, some of which are shown in this figure. Mutually beneficial interactions are represented by green arrows, while one-sided antagonistic interactions are represented by a bar-headed red line. The plant traits that underlie these interactions (such as nectar and the floral scent benzyl acetone) are shown in blue with the line thickness indicating the strength of the interaction.

FIGURE CREDIT: COYOTE TOBACCO BY STAN SHEBS (CC BY-SA 3.0); HUMMINGBIRD BY MDF (WIKIMEDIA COMMONS; CC BY-SA 3.0); HAWKMOTHS BY KELSEY JRP BYERS (CC BY 4.0).

Coyote tobacco attracts its pollinators with floral scent and rewards them with nectar. Kessler et al. studied these interactions using an approach that is innovative in a number of ways. First, they used RNA interference to silence the genes underlying the production of floral scent or nectar, either alone or in combination. This allowed them to evaluate specific floral traits in living plants, without too many confounding changes in other traits. Second, the approach is also unusual because few previous studies have combined plant-pollinator or plant–herbivore interactions and genetic manipulation in the study of floral scent (but see Kessler et al., 2008; Klahre et al., 2011; Kessler et al., 2013; Byers et al., 2015). Finally, it is also uncommon to combine field studies with more controlled greenhouse studies. This is important because while greenhouse studies can be more sensitive, their results do not always translate to the field (Obrycki and Tauber, 1984).

Pollinators are often classified into "guilds" of species that are presumed to interact with plants in similar fashions. However, little experimental work has studied the responses of different pollinator species *within* a guild. Kessler, Baldwin and colleagues address this issue, perhaps in an unforeseen way, by testing three different pollinators of coyote tobacco. Although *M. sexta* and *H. lineata* are both hawkmoths, they behave differently. When acting as a pollinator, *M. sexta* prefers wild-type plants to those lacking in scent or nectar or both, with all three alternatives being equally unattractive. *H. lineata*, on the other hand, treats wild-type plants and plants that lack scent or nectar the same, and prefers all three to plants that lack both scent and nectar. Hummingbirds, meanwhile, do not visit plants that lack nectar, and also appear to display a weak preference for plants that produce scent. This is perhaps unexpected because the flowers of coyote tobacco give off little scent during the day when the hummingbirds are foraging; hummingbirds also have a poor sense of smell and a limited ability to learn floral scent (Byers et al., 2015). These results – in particular, the fact that *M. sexta* and *H. lineata* behave differently, despite being members of the same guild – are also unexpected and argue for a more complex and nuanced picture of plant-pollinator interactions.

Kessler et al. found that *M. sexta* moths show different preferences when acting as pollinators compared to when they act as a floral antagonists. As a pollinator, *M. sexta* responds equally strongly to the loss of both scent and nectar. However, as an antagonist, this moth responds more strongly to the loss of nectar than it does to the loss of floral scent.

It is difficult to include multiple floral phenotypes and floral interactors in the study of plant-pollinator interactions, and as such this area remains largely unexplored. By addressing some of the related questions, Kessler et al. remind us of the value of an integrative approach. Their findings also suggest that future research in this area should consider whether model pollinators are representative of the real visitor community, and whether aspects such as learning play a role in these interactions. Flowers rarely occur alone, and thus considering the role of the surrounding floral community and background scents will also be important (Riffell et al., 2014). Research that combines floral scent and other phenotypes, their underlying genes, and their role in interactions with specific pollinators in a community context will, in the future, broaden our understanding of the field of plant–visitor interactions.
